# *Bacillus coagulans* alleviates hepatic injury caused by *Klebsiella pneumoniae* in rabbits

**DOI:** 10.1371/journal.pone.0317252

**Published:** 2025-01-10

**Authors:** Xiaoguang Chen, Wenjuan Wei, Fan Yang, Jianing Wang, Qiongxia Lv, Yumei Liu, Ziqiang Zhang

**Affiliations:** College of Animal Science and Technology, Henan University of Science and Technology, Luoyang, China; Tanta University Faculty of Agriculture, EGYPT

## Abstract

**Background:**

As an opportunistic bacterial pathogen, *Klebsiella pneumoniae* (KP) is prone to causing a spectrum of diseases in rabbits when their immune system is compromised, which poses a threat to rabbit breeding industry. *Bacillus coagulans* (BC), recognized as an effective probiotic, confers a variety of benefits including anti-inflammatory and antioxidant properties.

**Aim:**

The purpose of this study was to investigate whether dietary BC can effectively alleviate hepatic injury caused by KP.

**Methods:**

In this study, the rabbits were initially pretreated with varying doses of BC (1×10^6^, 5×10^6^, and 1×10^7^ CFU/g), followed by a challenge with KP at a concentration of 10^11^ CFU/mL. Liver tissues were harvested and processed for histological assessment using H&E and VG stains to assess structural alterations. Biochemical assays were employed to quantify the enzymatic activities of T-SOD and GSH-Px, as well as the MDA content. Furthermore, ELISA was utilized to detect the levels of inflammatory cytokine (IL-10, IL-6, IL-1β and TNF-α) and apoptotic-related gene (Bcl-2, Bax).

**Results:**

Morphological observation indicated that BC can effectively mitigate KP-induced hepatic sinusoidal dilatation and congestion, as well as ameliorate the degree of hepatic fibrosis. Further analysis showed that BC significantly lowered MDA level in KP-treated rabbits, while enhanced the activities of T-SOD and GSH-Px. Additionally, ELISA result showed that BC pretreatment significantly reduced the levels of pro-inflammatory cytokines TNF-a, IL-6, IL-1β and pro-apoptotic gene Bax, while increasing the levels of anti-inflammatory cytokine IL-10 and anti-apoptotic gene Bcl-2 in KP-treated rabbits.

**Conclusion:**

Above data indicate that BC supplementation effectively attenuated oxidative stress and inflammatory response induced by KP through augmenting the activities of antioxidant enzymes and diminishing the levels of pro-inflammatory factors. Furthermore, it reduced the Bax/Bcl-2 ratio in the liver, thereby inhibiting KP-induced apoptosis. The treatment group receiving 5x10^6^ CFU/g BC benefitted most from the protective effect.

## Introduction

Klebsiella is a genus of gram-negative bacillus within the Enterobacteriaceae family, mainly consisting of three subspecies: *Klebsiella odorata*, *Klebsiella rhinosclerotiorum*, and *Klebsiella pneumoniae* (KP) [1]. KP is a prominent opportunistic pathogen and exhibits the strongest pathogenicity of the three [2]. These short, thick and rod-shaped bacteria possess a thick capsule, numerous pilli, and lack spores and flagella. They are ubiquitous in the environment, particularly in soil, grains and water. They can colonize the intestinal, respiratory, and urogenital tracts of both humans and animals [3,4]. As an opportunistic pathogen, KP disseminates in hosts with compromised immunity. It can cause various diseases including systemic sepsis, liver abscess, encephalitis, hepatitis and mastitis in humans and animals [5]. All animals, regardless of breed, gender and age, are susceptible to these pathogenic bacteria. Young animals are particularly susceptible [6]. Studies have found that the incidence rate of weaned rabbits was about 30%, and the mortality rate was about 16%. At the onset of disease young rabbits show symptoms such as dyspnea, acute diarrhea, and abdominal distension. In weaned rabbits that were infected with KP and died it was found that most had significant mucoid catarrhal enteritis and punctate hemorrhage of cecal wall. A few cases showed constipation, mesenteric lymph node edema and splenomegaly [7]. Symptoms of disease that have been observed in New Zealand sea lions infected with KP and include diarrhea, suppurative arthritis, cerebellar hernia, lymphadenitis, and cellulitis [8,9]. Concurrently, the incidence of KP infections has escalated gradually, posing a formidable threat to the health of both humans and animals.

Many studies have found that KP mainly colonizes the intestine, leading to increased intestinal inflammation and intestinal microbial diversity disorders [10,11]. Once the intestinal epithelial cell barrier is destroyed, KP spreads into the blood and other vital organs such as liver [12]. The liver, an important organ responsible for metabolism, detoxification and immune response, plays a critical role in the clearance of pathogens like bacteria and viruses from the bloodstream and during this process KP can inflict hepatic damage, potentially leading to severe liver complications [13]. For instance, Das et al. isolated KP from the blood and liver of diseased fish and observed pathological phenomena such as necrosis, nuclear fragmentation and vacuolization of hepatocytes. Studies have indicated that when the immunity system of rabbits weakens, they become more susceptible to invasion by KP that could penetrate the intestinal barrier and invade and damage the liver. The resulting acute diarrhea and liver impairment can potentially lead to the death of rabbits [14]. KP can cause liver abscesses which are very difficult to treat and can lead to a mortality rate of 10 to 30% [15,16]. To date, few studies have been aimed at treating KP infection in rabbit livers.

In industrial application, the strategic utilization of antibiotics has been demonstrated to be effective in enhancing growth performance and reducing the incidence of diseases. However, the extensive and prolonged use of antibiotics has resulted in heightened drug resistance in KP. Furthermore, the overuse of antibiotics causes increased levels of drug residues, posing a risk to human health. In response, China has instituted a ban on the inclusion of antibiotics in animal feed. As a result, the need to explore and develop new products as viable alternatives to antibiotics has become increasingly urgent.

Probiotics have the advantage of non-toxic side effects, no residue, no drug resistance, no environmental pollution, and low cost. Among them, *Bacillus coagulans* (BC) which is known as the "king of probiotics", is considered an ideal antibiotic substitute in the feed industry [17,18]. BC is not an inherent bacterium in the gut, but a microorganism capable of forming spores. It has been demonstrated that BC, when incorporated into rabbit feed, not only survives but also thrives within the intestinal tract. Other studies have also shown that spores formed by *Bacillus* subtilis feed additives in the gastrointestinal tract have functional characteristics that combine with nutritional cells [19,20]. Beyond its role as a dietary supplement ingredient, BC has emerged as an active component in many pharmaceuticals that have entered the global market, demonstrating their clinical efficacy [21]. Studies have shown that the addition of BC to feed can modulate the composition of gut microbiota, improve the intestinal microecological environment, bolster intestinal function, facilitate intestinal development, and enhance both immune response and production performance of animals [22–24]. Specifically, the inclusion of 2~5×10^6^CFU/g of BC in the feed of Guangxi yellow chickens has been shown to significantly increase their daily weight gain, optimize feed conversion rate, and improve meat quality [25]. One study found that BC could reduce diarrhea and enhance growth in piglets [26]. Adding BC to the diets of growing pigs was found to be a substitute for aureomycin and maintained their normal growth [27]. BC can also enhance feed conversion efficiency by improving the equilibrium of gut microbiota, thereby fostering the growth of broiler chickens [28]. Also, studies have shown that BC can alleviate inflammation in the body, enhance the absorptive surface area of villi to facilitate nutrient absorption, and effectively promote the rapid restoration of the intestinal microbiota, thereby contributing to gut health [29]. Furthermore, studies have elucidated that BC can antagonize endotoxin-triggered inflammatory responses by inhibiting the secretion of pro-inflammatory cytokines such as IL-8 and increasing the secretion of anti-inflammatory cytokines like IL-10 [30]. Additionally, BC exhibits robust antioxidant capability, protecting the host from oxidative stress induced by metals including mercury and chromium [22,23].

Given the above, the question arises of whether BC can mitigate hepatic injury in rabbits induced by KP. To date, there have been limited reports on this subject. To address this issue, the present study pre-treated experimental rabbits with different dosages of BC prior to KP challenge. By employing a range of methodologies, such as histological examination and biochemical assays, we aimed to elucidate the effect of BC on hepatic injury induced by KP, thereby providing a possible method for KP prevention and control in rabbit husbandry.

## Materials and methods

### Chemicals and reagents

The total superoxide dismutase (T-SOD) Assay Kit, glutathione peroxidase (GSH-Px) Assay kit, malondialdehyde (MDA) Content Assay Kit and BCA Protein Quantification Kit utilized in this experiment were all purchased from Wuhan Servicebio Technology Co., Ltd. The enzyme-linked immunosorbent assay (ELISA) kits for Interleukin-10 (IL-10), Interleukin-1β (IL-1β), Interleukin-6 (IL-6), Tumor necrosis factor-α (TNF-α), B-cell lymphoma-2 (Bcl-2) and Bcl-2-associated X protein (Bax) were all obtained from Nanjing SenBeiJia Biological Technology Co., Ltd. The hematoxylin-eosin (H&E) staining kit was purchased from Beyotime Biotechnology. The Van Gieson ’s (VG) staining kit was sourced from Servicebio.

### Animals and treatments

**Fig 1:**
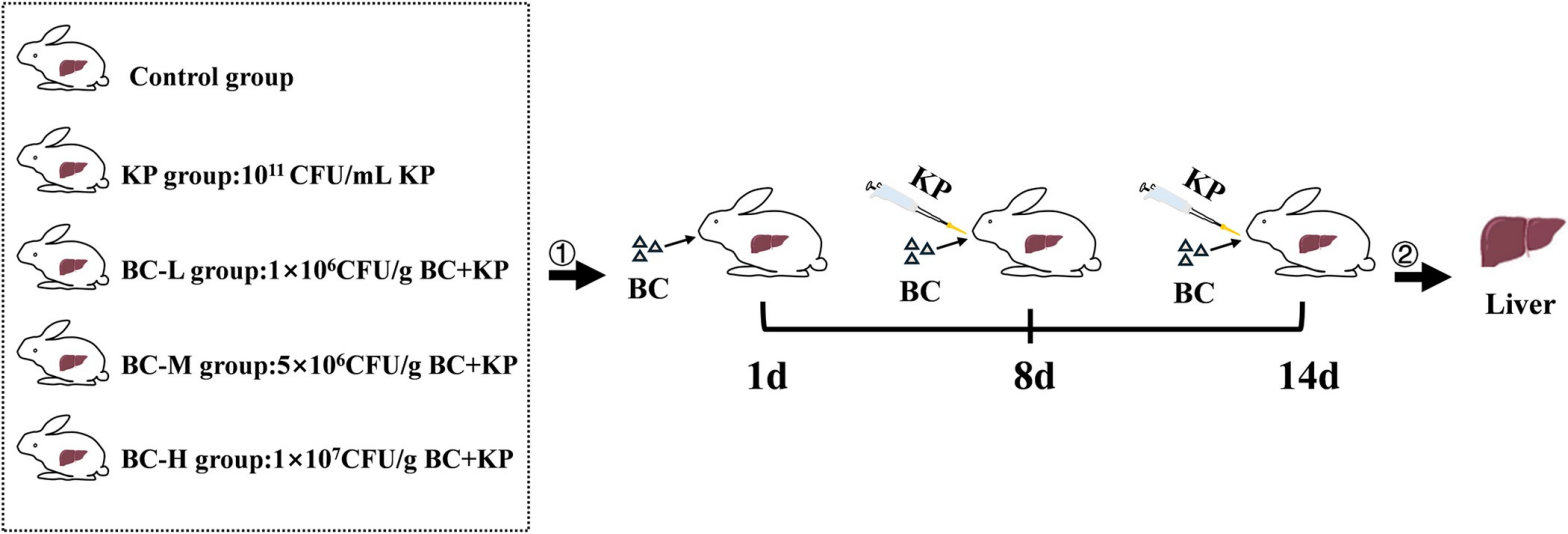
Flow chart of experimental design for constructing KP model. KP: *Klebsiella pneumoniae*; BC: *Bacillus coagulans*.

Fifty 35±1-day-old healthy rabbits used in this experiment were provided by the Animal Experiment Centre of Henan University of Science and Technology. After being acclimatized for 7 days (21~25°C, 50~70% relative humidity), the animals were randomly assigned into 5 groups (10 rabbits each) as follows: control, KP, and BC low, medium, and high-dose groups. During the feeding process, each rabbit was fed 200g of basic diet per day but was allowed to drink water freely. During days 1–14 of the experiment, the rabbits in three BC groups were fed with basal diets that contained BC concentrations of 1×10^6^, 5×10^6^, and 1×10^7^ CFU/g, respectively, and the control and the KP groups were fed with the regular diets. From day 8 to 14 of the experiment, animals in the KP group and the three BC groups were orally gavage with 4 mL of KP bacterial suspension (1×10^11^ CFU/mL), once daily for 7 consecutive days, while control group was gavage with an equivalent volume of sterilized saline during this period. Animals were sacrificed by an intravenous overdose of 3% pentobarbital sodium 24 h after the last dose (Fig 1) [25]. The KP was isolated and purified from the infected rabbits, and BC was purchased from Henan Anmai Kang Biotechnology Co., LTD. All animal care and experimental procedures were conducted in strict adherence to the institutional guidelines as well as national regulatory standards, and they were formally approved by the Institutional Animal Care and Use Committee of Henan University of Science and Technology (China) (No.20190619024).

### Sample collection

Twenty-four hours following the final challenge with KP, all the animals were euthanized in accordance with the method mentioned above. Then, the liver tissues were rapidly harvested and rinsed in cold (4°C) physiological saline, with some samples fixed in 4% polyformaldehyde for histopathological examination and the remaining tissues stored at -20°C for subsequent biochemical analysis [31].

### Histomorphology examination

Following fixation of the freshly obtained liver tissue samples in a 4% paraformaldehyde solution, the samples were dehydrated in a graded alcohol series. And the dehydrated tissues were treated with xylene transparent agent to remove alcohol. The liver tissue was then impregnated with melted paraffin. Then the impregnated tissue was put into a mold where new paraffin was added. The paraffin was then solidified by cooling. Finally, the paraffin-embedded tissue block was sectioned into thin slices with a thickness of 5μm by a microtome, and then these sections underwent dewaxing [32].

Three samples were randomly chosen from each group for paraffin section preparation and H&E staining. The tissue was stained using H&E using standard procedures (Beyotime Biotechnology; C0105). Finally, the sections were mounted with neutral resin, and the images were using light microscopy (Nikon; ECLIPSE C1) [33]. Three distinct regions were randomly selected from each slice within each group for the pathological evaluation of liver tissue samples [34], and the representative slice from each group was chosen and included in the “Results” section.

Three samples were randomly chosen from each group for paraffin section preparation and Van Gieson’s (VG) staining. Liver tissue was also stained using Van VG stain according to standard procedure (Service bio; G1046). These sections were mounted with neutral resin and the images captured using light microscopy (Nikon; NIKONECLIPSEE100) [35]. The METAVIR scoring system was used for evaluating the degree of liver fibrosis in three randomly selected regions of each slice within each group: 0, no fibrosis; 1, portal fibrosis without septa; 2, few septa; 3, numerous septa without cirrhosis; 4, cirrhosis [36]. Finally, the representative slice from each group was selected and highlighted in the “Results” section.

### Analysis of antioxidants and oxidative biomarkers

The frozen tissues were retrieved from -80°C freezer. 50mg of each sample was accurately weighed and then homogenized in 0.5mL of cold (4°C) PBS buffer using a tissue homogenizer for 5min. The 10% homogenized solution was centrifuged at 3000 rpm for 10 min at 4°C to obtain a supernatant that was rapidly reserved at -20°C for further analysis. A standard curve MDA and total protein was drawn and the activity of T-SOD and GSH-Px in the liver tissue samples were measured by spectrophotometer (Shenzhen Leidu Life Science; RT-6100) using the corresponding detection kits. All data were normalized to the total protein concentration of each sample. The MDA content is represented by μmol/gprot protein, and the activities of T-SOD and GSH-Px are represented by U/mgprot protein [31].

### Detection of inflammatory factors

The homogenization of liver tissue and the preparation of its supernatant were performed according to previously described procedures. ELISA was carried out according to the instructions of the kit, and the standard curve was operated and drawn according to the method of the kit, a standard curve was drawn and the levels of IL-6, TNF-a, IL-1β and IL-10 in the liver homogenate were detected by spectrophotometer (Shenzhen Leidu Life Science; RT-6100) [37]. The levels of the cytokines are expressed as nanograms per liter (ng/L).

### Measurement of apoptosis-related factors

The levels of Bcl-2 and Bax in the tissue homogenates were quantified using ELISA according to the protocols specified in the kit instructions [38]. The absorbance values were recorded at the wavelength of 450 nm using a microplate reader (Shenzhen Leidu Life Science; RT-6100). A standard curve was plotted based on the standard concentrations to calculate the concentrations of Bcl-2 and BAX that are expressed in ng/mL.

### Statistical analysis

The collected data were analyzed using one-way analysis of variance (ANOVA) and least squares tests within the SPSS 20.0 statistical software. The results were presented as mean values ± standard deviation (SD), and the differences among groups were considered as statistical significance at levels of *p* < 0.05.

## Results

### BC attenuates liver tissue structural damage induced by KP in rabbits

**Fig 2:**
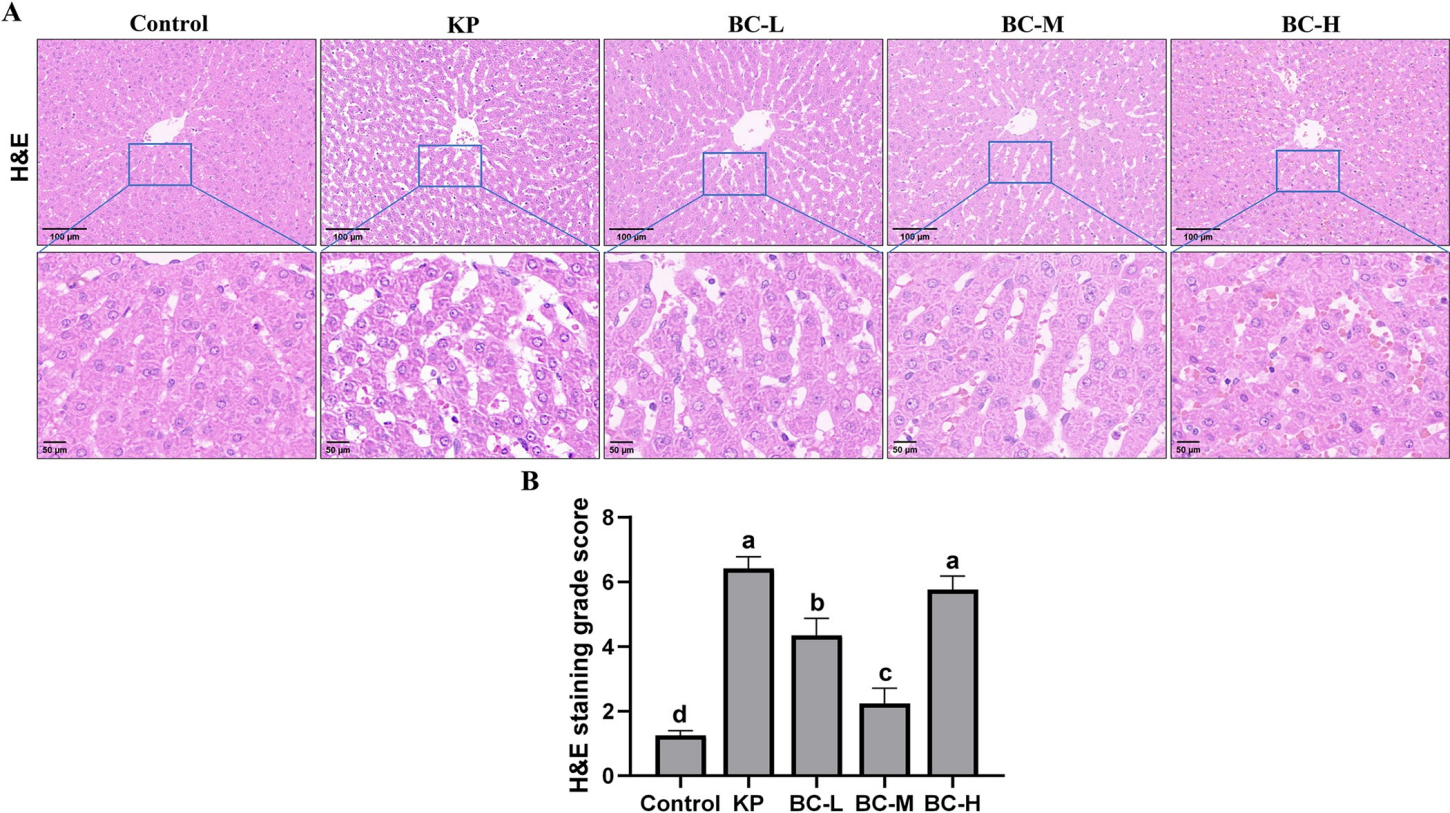
Morphological and structural examination of the liver. (A): The representative images of H&E-stained liver sections. (B): H&E staining grade score. Control: Control group; KP: *Klebsiella pneumoniae* group; BC-L: Low-dose *Bacillus coagulans* +KP group; BC-M: Medium-dose *Bacillus coagulans* +KP group; BC-H: High-dose *Bacillus coagulans* +KP group. Data represented as mean ± SD, n  = 5; a~d different lowercase letters indicate significance, same letters mean insignificant difference between groups (*p*>0.05), while different letters indicate significant difference between groups (*p*<0.05).

Histomorphology analysis was conducted utilizing H&E staining. Histopathologic evaluation was performed in accordance with the MAFLD scoring system, as depicted in (Fig 2A and 2B). In comparison to the control group, the KP group demonstrated a disrupted arrangement of hepatic cord, blurred contours, severe dilation and congestion of hepatic sinusoids, along with cellular membrane rupture and vacuolation. The pathology score was significantly increased in the KP group when compared to the control (*p*<0.05). Compared to KP group, the BC low- and medium-dose groups, especially medium-dose group exhibited relatively ordered hepatic cords with less dilation of central veins and hepatic sinusoids, obviously reduced congestion, clear cellular membrane boundaries, minimal vacuolation and significantly decreased pathology scores (*p*<0.05). However, the pathological changes in the BC high-dose group were like those in the KP group, with relatively prominent congestion in the central veins and hepatic sinusoids, blurred cellular membrane boundaries with no significant difference in the pathology score compared with the KP group (*p*>0.05).

**Fig 3:**
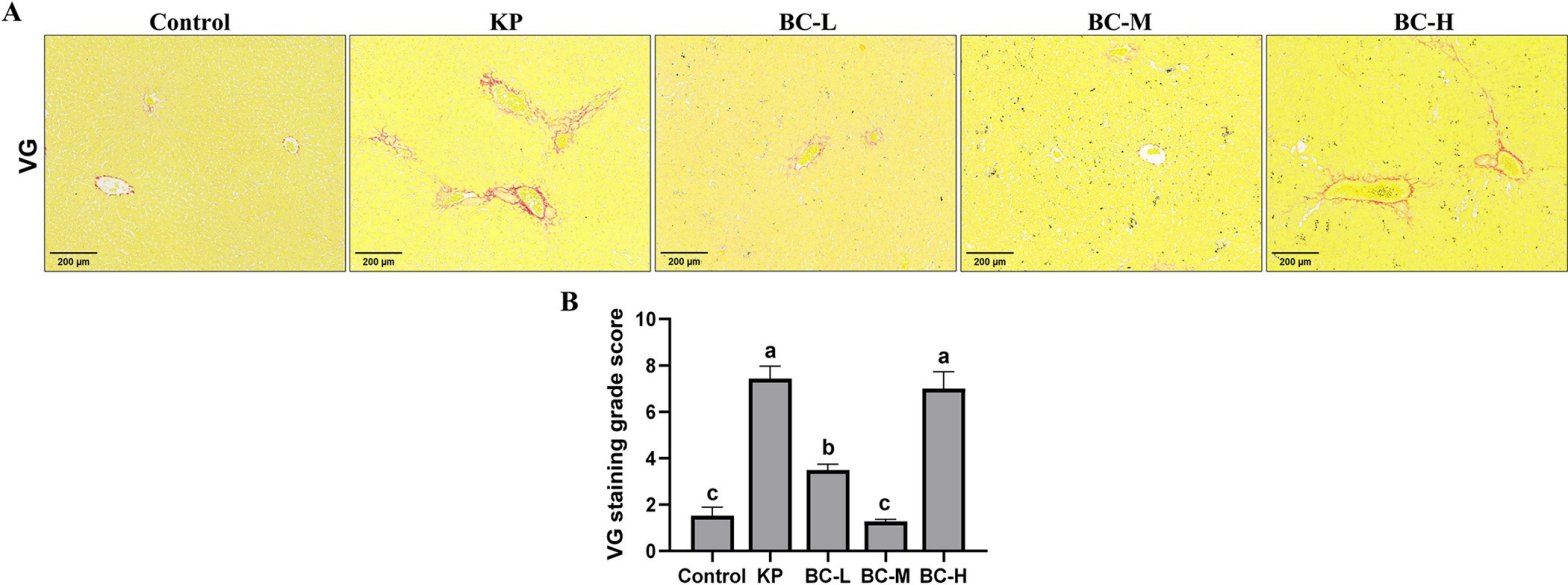
Detection of liver fibrosis degree. (A): The representative images of VG-stained liver sections. (B):VG staining grade score. Control: Control group; KP: *Klebsiella pneumoniae* group; BC-L: Low-dose *Bacillus coagulans* +KP group; BC-M: Medium-dose *Bacillus coagulans* +KP group; BC-H: High-dose *Bacillus coagulans* +KP group. Data represented as mean ± SD, n  = 5; a~d different lowercase letters indicate significance, same letters mean insignificant difference between groups (*p*>0.05), while different letters indicate significant difference between groups (*p*<0.05).

Furthermore, hepatic collagen fiber formation was determined using VG staining and the degree of liver fibrosis was assessed using METAVIR scoring system. As shown in Fig 3A, no significant fibrous tissue hyperplasia was observed in the control group. However, after the administration with KP, there was marked increase in fibrous tissue hyperplasia, characterized by fiber crosslinking that disrupted the structure of hepatic lobule. Statistical analysis revealed a significant elevation in liver fibrosis grading in the KP group relative to the control group (*p*<0.05). After pretreatment with BC, both the low- and medium-dose groups, especially the medium dose group, demonstrated a significant reduction in fibrous tissue and a substantially decrease in liver fibrosis scores compared to the KP group (*p*<0.05). However, in the high-dose BC group, there was hyperplasia of hepatic fibrous tissue, formation of fibrous septa, and disruption of lobule structure, with no significant difference in the liver fibrosis score when compared with the KP group (*p*>0.05) (Fig 3B).

### BC improves hepatic oxidative status induced by KP in rabbits

**Fig 4:**
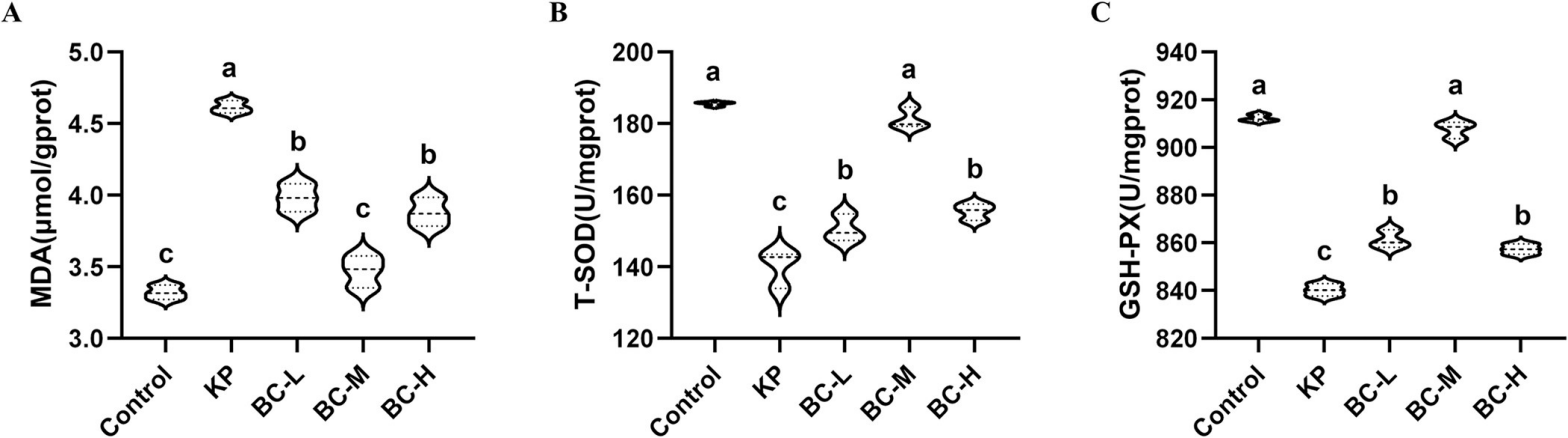
Effect of BC on hepatic oxidative stress in KP-treated rabbits. (A): Malondialdehyde (MDA) content. (B): Total superoxide dismutase (T-SOD) activity. (C): Glutathione peroxidase (GSH-Px) activity. Control: Control group; KP: *Klebsiella pneumoniae* group; BC-L: Low-dose *Bacillus coagulans* +KP group; BC-M: Medium-dose *Bacillus coagulans* +KP group; BC-H: High-dose *Bacillus coagulans* +KP group. Data represented as mean ± SD, n  = 5; a~d different lowercase letters indicate significance, same letters mean insignificant difference between groups (*p*>0.05), while different letters indicate significant difference between groups (*p*<0.05).

To determine whether BC provided a protective effect against KP-induced hepatic injury via its antioxidant properties, biochemical test kits were utilized to assess the level of oxidative biomarker MDA and the activities of enzymatic antioxidants T-SOD and GSH-Px in hepatic tissues. As shown in (Fig 4), compared with the control group, MDA level in KP group was significantly increased by 39.0% (*p*<0.05) (Fig 4A), while GSH-Px and T-SOD activities were significantly decreased by 7.9% and 24.5% (*p*<0.05) respectively (Fig 4B and 4C). When compared to the KP group, the content of MDA was significantly decreased by 13.7% the low dose group, 24.8% in the medium dose group and 15.9% in the high dose group (*p*<0.05). The activities of T-SOD and GSH-Px were significantly increased (*p*<0.05) in the animals receiving the BC-supplemented diet. Specifically, the T-SOD activity increased 7.5% in the low dose group, 29.4% in the medium dose group and 11.01% in the high dose group (*p*<0.05). GSH-Px also increased respectively by 2.5%, 8.0% and 2.0% (*p*<0.05).

### BC mitigates hepatic inflammation induced by KP in rabbits

**Fig 5:**
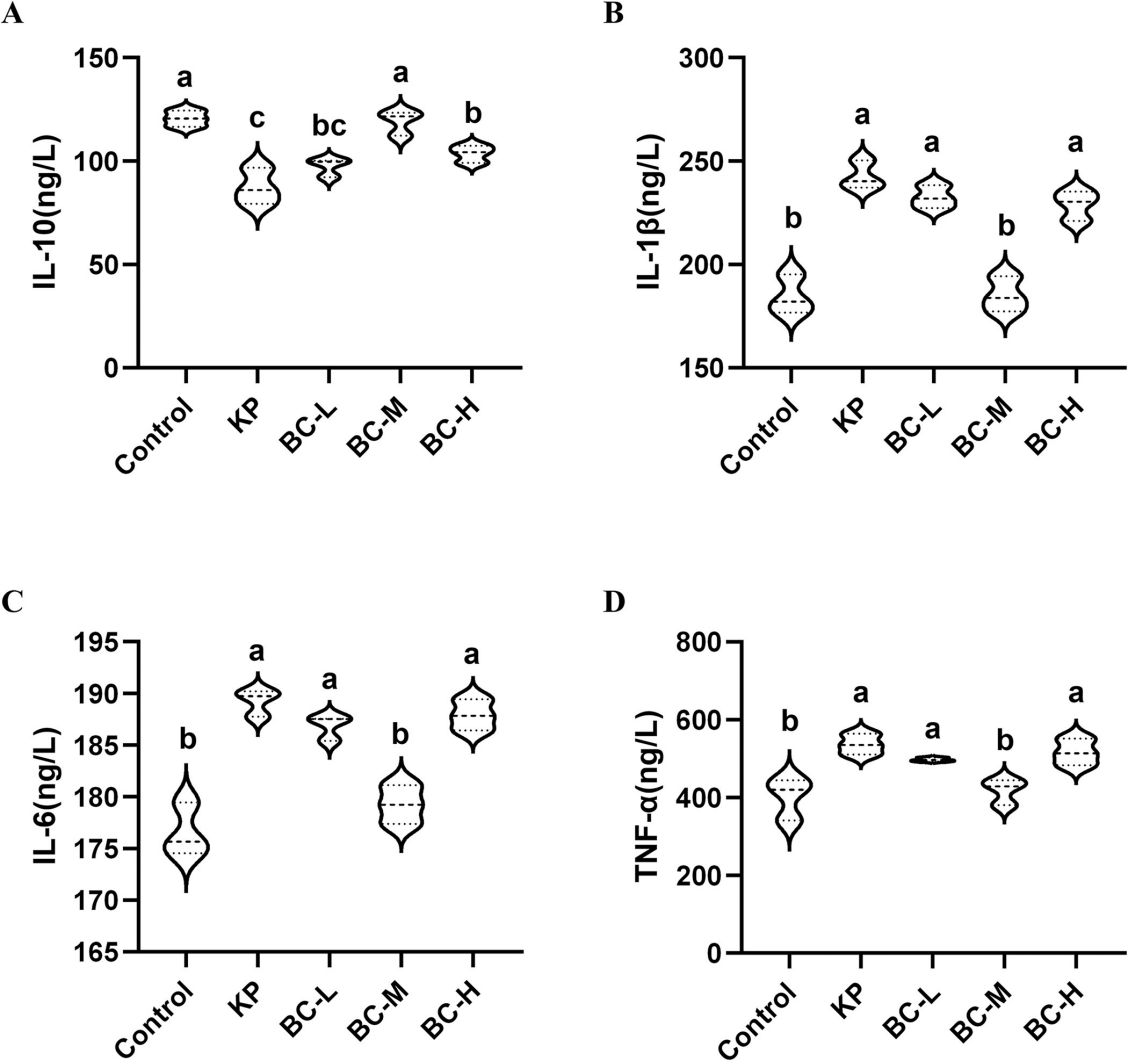
Effect of BC on inflammatory cytokines in KP-treated rabbit liver. (A): Interleukin- 10 (IL-10) level. (B): Interleukin- 1β (IL-1β) level. (C): Interleukin-6 (IL-6) level. (D): Tumor necrosis factor-α (TNF-α) level. Control: Control group; KP: *Klebsiella pneumoniae* group; BC-L: Low-dose *Bacillus coagulans* +KP group; BC-M: Medium-dose *Bacillus coagulans* +KP group; BC-H: High-dose *Bacillus coagulans* +KP group. Data represented as mean ± SD, n  = 5; a~d different lowercase letters indicate significance, same letters mean insignificant difference between groups (*p*>0.05), while different letters indicate significant difference between groups (*p*<0.05).

Oxidative stress can result in an elevation of intracellular oxygen free radicals and other reactive oxidant species, subsequently triggering inflammatory response. In the present study, the levels of IL-6, TNF-α, IL-1β, and IL-10 in rabbit hepatic tissues were measured using the ELISA method. As shown in Fig 5 the level of IL-10 was significantly decreased by 27.5% (*p*<0.05) (Fig 5A) when compared to the control group while the levels of pro-inflammatory cytokines IL-1β, IL-6, and TNF-α were increased by 31.4%, 7.2%, and 33.5%, respectively (*p*<0.05) (Fig 5B–5D). When compared with values obtained from the medium dose BC group, the IL-10 level was significantly while the Il-1β, IL-6 and TNF-α levels were significantly decreased (*p*<0.05).

### BC alleviates KP-induced hepatic apoptosis in rabbits

**Fig 6:**
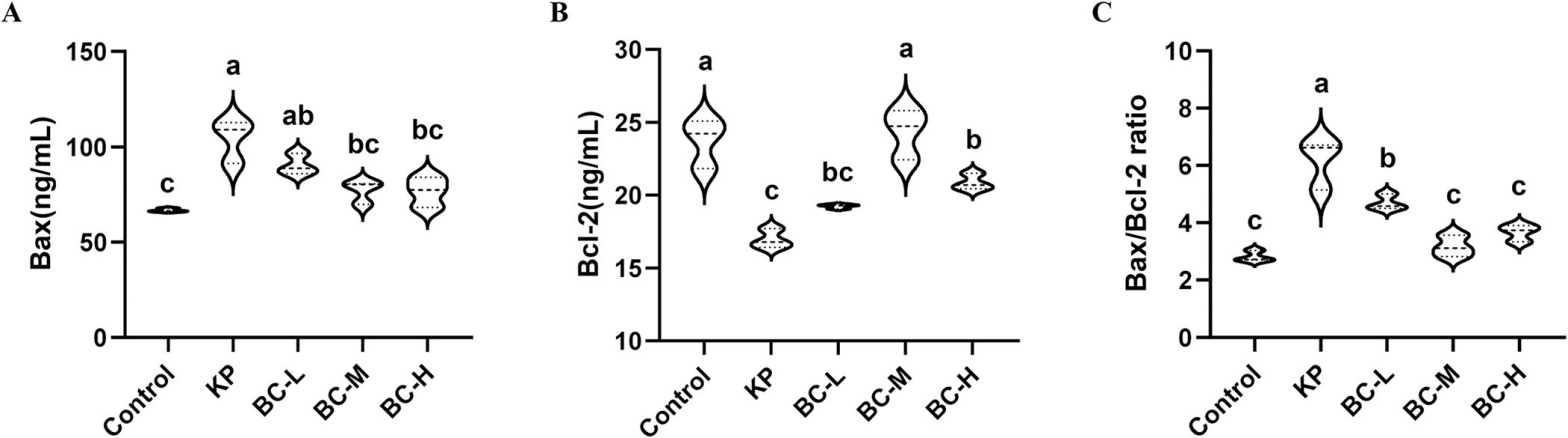
Effect of BC on apoptosis of rabbit liver cells treated by KP. (A): Bcl-2-associated X protein (Bax) level. (B): B-cell lymphoma-2 (Bax) level. (C): Bax/Bcl-2 ratio. Control: Control group; KP: *Klebsiella pneumoniae* group; BC-L: Low-dose *Bacillus coagulans* +KP group; BC-M: Medium-dose *Bacillus coagulans* +KP group; BC-H: High-dose *Bacillus coagulans* +KP group. Data represented as mean ± SD, n  = 5; a~d different lowercase letters indicate significance, same letters mean insignificant difference between groups (*p*>0.05), while different letters indicate significant difference between groups (*p*<0.05).

The contents of the apoptosis-associated protein Bcl-2 and Bax in rabbit hepatic tissues were measured using ELISA. As shown in Fig 6, compared with the control group, the level of pro-apoptotic gene Bax was significantly increased (*p*<0.05) and the level of anti-apoptotic gene Bcl-2 was significantly decreased (*p*<0.05) in the KP-treated group, whereas the level of gene Bax was significantly decreased and the level of gene Bcl-2 was significantly increased (*p*<0.05) after pretreatment with medium- and high-dose BCs (Fig 6A and 6B); In addition, compared with the control, the ratio of Bax/Bcl-2 in the KP-challenged group was significantly increased (*p*<0.05), while significantly decreased by 23.8%, 48.5% and 40.6% after pretreating with three different doses of BC (*p*<0.05) (Fig 6C).

## Discussion

*Klebsiella pneumoniae* (KP), a gram-negative bacterium, is ubiquitously present in the natural environment [39,40]. When the animal’s immune system is compromised, this opportunistic pathogen proliferates and spreads rapidly in the body, breaching the intestinal barrier, and thus causes extraintestinal infections. The liver is recognized as the most affected extraintestinal organ [12]. Hepatic invasion by KP can result in liver damage, and in more severe cases, may lead to pyogenic liver abscesses [41]. It has been reported that 1×10^4^ CFU/mL of KP treatment could lead to vacuolar degeneration of hepatocytes, accompanied by severe tissue necrosis, interstitial edema, and inflammatory cell infiltration in the murine liver [41,42], which is congruent with our observations of KP-induced congestion and swelling of hepatic sinusoids, hepatocyte disruption and cytoplasmic vacuolation in rabbits. It has been confirmed that chronic hepatic injury can precipitate liver fibrosis [43]. Accordingly, Van Gieson (VG) staining was used to detect hepatic fibrosis in this study. and the results showed that KP challenge could lead to a significantly increased degree of hepatic fibrosis in rabbits, which is just consistent with previous research findings that KP infection could cause fibrosis damage to liver organoids [43], thereby re-affirming KP’s detrimental impact on hepatic tissues. *Bacillus coagulans* (BC), a gram-positive bacillus and a widely utilized probiotic in animal husbandry, has been shown to significantly alleviate the hepatic sinusoidal disorders, vacuolar degeneration, and inflammatory damage induced by chromium (Cr) exposure [23]. Supplementing BC can markedly reduce ethanol-induced liver steatosis in mice [44]. However, the efficacy of BC in mitigating KP-induced hepatic injury remains unclear. Our study indicated that BC pretreatment effectively ameliorated KP-induced hepatocyte swelling, disruption, vacuolation, and fibrosis in rabbits, suggesting that an optimal dosage of BC could reduce hepatic injury in KP-challenged rabbits. Interestingly, we found that a high dose of BC has a more moderate protective effect on KP-induced liver injury compared to low and medium doses. We speculate that this diminished effect at high dose might be due to the poor taste of the feed in the high-dose group leading to an insufficient intake of BC, which thereby resulted in the suboptimal effect of the high-dose group. Nevertheless, the mechanism underlying the better effect of the medium dose than the high dose remains unknown and requires further investigation.

Oxidative stress is a critical mediator of hepatic injury [45]. Under normal physiological conditions, a precise equilibrium is maintained between the production of reactive oxygen species (ROS) and the antioxidant defense system, which includes enzymes such as T-SOD, GSH-Px, CAT, and non-enzymatic antioxidants like GSH. This balance is essential for preserving intracellular redox homeostasis and safeguarding cells against oxidative stress. Nonetheless, oxidative damage ensues when ROS production surpasses the regulatory capacity of antioxidant system [46]. Documents have reported that KP could induce oxidative stress, causing tissue injury in various organs, including the intestine, lung, and liver [47–49]. In our study, we observed that intragastric administration of KP significantly reduced the enzymatic activities of key antioxidants (T-SOD, GSH-Px) in rabbit hepatic tissue, concurrently increased the level of MDA. Research has indicated that BC is instrumental in alleviating oxidative stress in the intestine and liver, particularly in the context of toxic metal exposure, such as cadmium and mercury [50,51]. Consistent with these findings, our result suggested that a specific dosage of BC, notably medium dose, effectively counteracted KP-induced oxidative stress in the rabbit liver, albeit the precise mechanism underlying this effect warrant further investigation.

It has been established that oxidative stress often occurs concurrently with inflammatory response and their mutual interaction further exacerbates tissue and cellular damage [52,53]. KP has been documented to trigger inflammatory response, resulting in damage to the liver, lung and digestive tract in animals [54–56]. Our study found that KP administration significantly upregulated the levels of pro-inflammatory cytokines IL-1β, IL-6 and TNF-α, while inhibiting the production of anti-inflammatory cytokine IL-10. Previously studied indicate BC alleviates the inflammatory responses in various tissues, including the intestine, ileum, and muscle, triggered by pathogens such as *Salmonella*, and Lipopolysaccharide (LPS) [57–59]. Our results showed that pretreatment with varying doses of BC, particularly the moderate dose, led to a pronounced downregulation of the three pro-inflammatory cytokines with a significant upregulation of IL-10, implying BC’s potential to ameliorate KP-induced inflammation in the rabbit liver. This is in line with a previous study that determined that BC can significantly decrease the levels of cytokines TNF-α and IL-1β and effectively alleviate hepatic inflammatory injury in mice [44]. We also found that the medium dose of BC is more effective than the high and low doses at reducing KP triggered inflammation. Considering other results from this study, the low dose (1×10^6^ CFU/g) BC in this study did not achieve the desired effect.

A decline in antioxidant capacity not only triggers inflammatory responses but also results in cellular damage due to cellular damage due to the inability to promptly eliminate reactive oxygen species (ROS), ultimately initiating cell apoptosis. The study showed that KP infection, disrupted the equilibrium between oxidation and anti-oxidation and induced apoptosis of lung epithelial cell in mice [60]. Liver cell apoptosis triggered by KP is dependent on the Bcl-2 family-mediated apoptosis pathway [61], and another study found that the addition of BC alleviated the hepatocyte apoptosis in rats by up-regulating Bcl-2 gene, and down-regulating Bax and Caspase-3 genes [62]. In our study, KP infection significantly decreased Bcl-2 levels and increased Bax levels, thereby elevating the Bax/Bcl-2 ratio, which causes mitochondrial release of cytochrome C (Cyto-C) and subsequently hepatocyte apoptosis in rabbits. Supplementation of BC significantly diminished the harmful effects of Enterotoxigenic Escherichia coli (ETEC) on the intestinal tract in piglets by reducing oxidative stress and cell apoptosis [63]. Similarly, our results indicated that a medium-dose BC could inhibit KP-induced cell apoptosis by suppressing the Bax/Bcl-2 ratio in the liver of rabbits. Due to the above results indicate that a medium dose of BC effectively mitigates liver damage induced by KP via reducing oxidative stress, and apoptosis in rabbits.

## Conclusion

**Fig 7:**
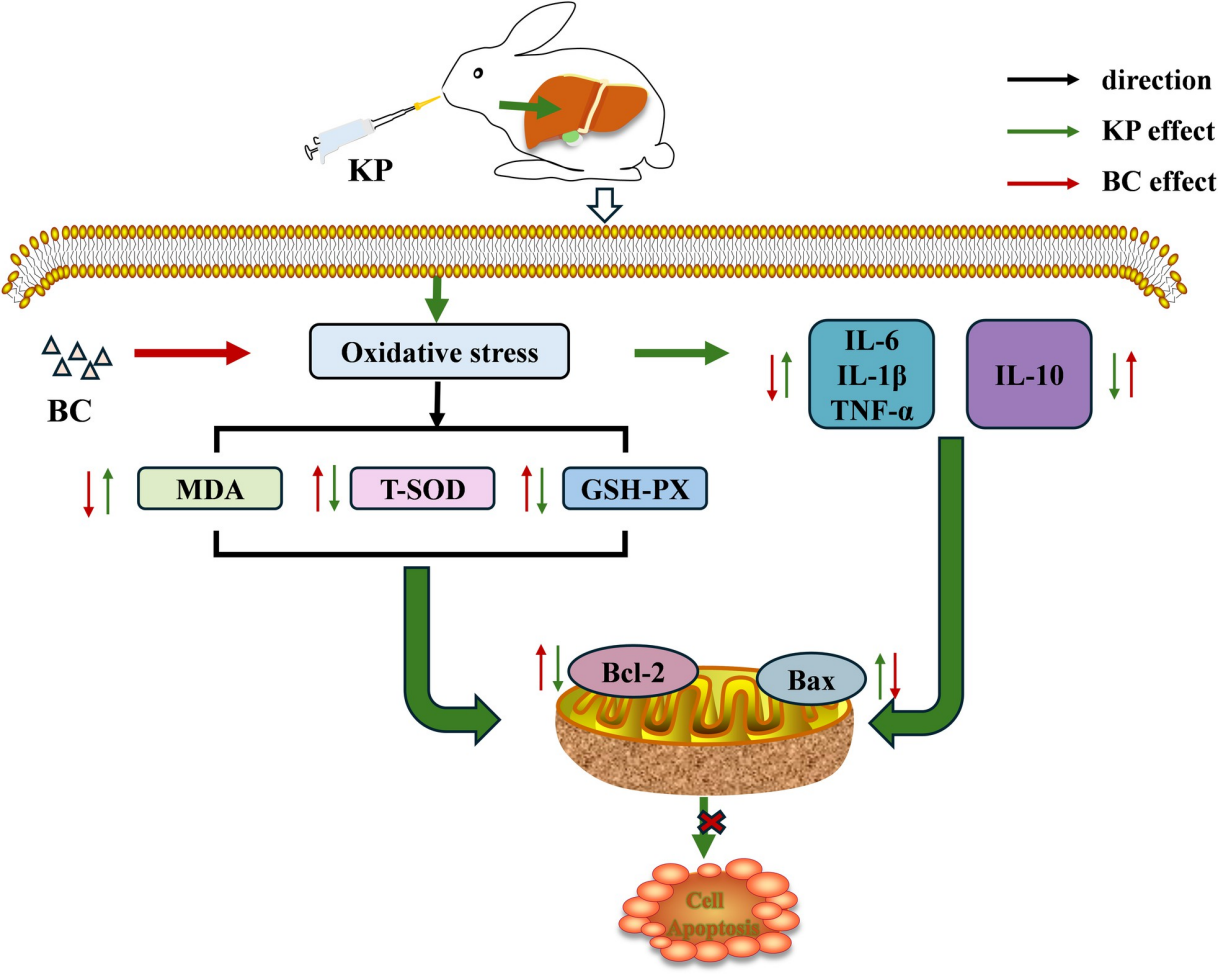
Schematic diagram of the protective effect of BC on hepatic injury induced by KP. BC alleviates liver damage induced by KP by reducing oxidative stress, inflammatory response, and apoptosis in the liver. KP: *Klebsiella pneumoniae*; BC: *Bacillus coagulans*; MDA: Malondialdehyde; GSH-Px: Glutathione peroxidase; T-SOD: Total superoxide dismutase; IL-6: Interleukin-6; IL-1β: Interleukin-1β; TNF-α: Tumor necrosis factor-α; IL-10: Interleukin-10; Bcl-2: B-cell lymphoma-2; Bax: Bcl-2 related X.

In summary, the incorporation of BC into rabbit feed has been demonstrated to effectively mitigate oxidative stress induced by KP and to attenuate inflammatory damage. This is achieved by enhancing the activities of antioxidant enzymes and reducing the levels of pro-inflammatory cytokines. Additionally, BC inhibits KP-induced apoptosis, potentially by lowering the Bax/Bcl-2 ratio in the liver (Fig 7). Adding 5×10^6^ CFU/g BC to the feed showed a significant protective effect on the liver in rabbits. Our results point to a possible application for the use of BC in the prevention and control of KP in rabbit husbandry. The precise mechanism of BC alleviating KP-induced hepatic injury in rabbits needs to be explored by further experiments, which will be the focus of our next study.

## Supporting information

S1 Table The data supports the findings of this study. (XLSX)

S2 Table Abbreviations used in this paper. (XLSX)
